# Harnessing Ribonucleoprotein Granule Biology for Cancer Therapy: The Central Role of Protein Modifications

**DOI:** 10.34133/research.1133

**Published:** 2026-02-18

**Authors:** Dengxiong Li, Qingxin Yu, Ruicheng Wu, Koo Han Yoo, Dilinaer Wusiman, Jie Wang, Qi Zhang, Dechao Feng

**Affiliations:** ^1^ Department of Urology, The First Affiliated Hospital of Zhejiang Chinese Medical University (Zhejiang Provincial Hospital of Chinese Medicine), Hangzhou, China.; ^2^ Department of Pathology, Zhejiang University School of Medicine, Research Unit of Intelligence Classification of Tumor Pathology and Precision Therapy, Chinese Academy of Medical Sciences (2019RU042), Hangzhou 310058, Zhejiang, China.; ^3^ Department of Pathology, Peking Union Medical College Hospital, Chinese Academy of Medical Sciences and Peking Union Medical College, Beijing 100730, China.; ^4^ Urology & Nephrology Center, Department of Urology, Zhejiang Provincial People’s Hospital (Affiliated People’s Hospital), Hangzhou Medical College, Hangzhou, Zhejiang, China.; ^5^ Division of Surgery & Interventional Science, University College London, London W1W 7TS, UK.; ^6^ Department of Urology, Kyung Hee University, Seoul, South Korea.; ^7^ Purdue Institute for Cancer Research, Purdue University, West Lafayette, IN 47907, USA.

## Abstract

Aberrant expression or dysfunction of ribonucleoprotein (RNP) granules usually contribute to cancer initiation, progression, and therapeutic response. In the process, protein modification importantly mediates the interactions between cancer and RNPs/associated proteins. Therefore, we tried to summarize and discuss the complex interactions between RNPs and various protein modifications in cancers, facilitating the clinical translation of RNP-based therapies. Studies have shown that RNPs can directly undergo phosphorylation, ubiquitination, SUMOylation, methylation, crotonylation, and acetylation, but no studies have reported glycosylation, fucosylation, or PARylation on RNPs. These modifications can competitively occur on RNPs, affecting the RNP granule expression and function. Cancer cells, immune cells, and stromal cells can undergo RNP granule modifications, consequently mediating current treatment efficacy. These results provide the basis of RNP granule-based antitumor therapy. However, the structural complexity of RNPs and limited research depth pose significant key for clinical translation.

## Introduction

Currently, cancer continues to be a major global cause of mortality, creating substantial societal burdens [1,2]. The number of new cancer cases increases from 19.3 million in 2020 to 20 million in 2022 [3,4]. Although extensively developed, existing treatments still lack satisfactory efficacy. With robotic assistance, surgery is evolving toward minimally invasive and high-precision approaches [5,6]. Radiotherapy, chemotherapy, and targeted therapy have been applied in clinical practice and bring survival benefits to patients with cancer [7]. Furthermore, immunotherapy, including anti-programmed cell death protein 1 (anti-PD-1),anti-programmed cell death-ligand 1 (anti-PD-L1), and chimeric antigen receptor T cell therapy, is emerging as an effective treatment to manage cancer in clinical practice [8,9]. However, surgery remains traumatic for cancer patients and carries risks of failure [10]. Drug and radiotherapy resistance are usually reported in clinical cancer management [11]. Of these, tumor microenvironment (TME), including cells and stromal elements, substantially influences cancer therapy resistance [12,13]. Immunotherapy shows a low response rate due to the immune evasion in the TME [14]. The treatment resistance provides poor survival benefits to patients with cancer, but brings heavy economical, mental, and social burdens to them. Consequently, researchers are exploring novel therapeutic targets and biomarkers to enable personalized cancer treatment strategies [15].

Ribonucleoprotein (RNP) granules are intracellular RNA–protein assemblies without membrane and formed through RNA–RNA, RNA–protein, and protein–protein interactions between RNPs [16]. RNPs usually include small nucleolar RNP (snoRNP), small nuclear RNP (snRNP), heterogeneous nuclear RNP (hnRNP), cytosolic processing bodies, nuclear Cajal bodies, and paraspeckles [17,18]. Of these, the hnRNP family comprises more than 30 members, including hnRNP A1-U [19]. Moreover, snoRNPs, comprising a snoRNA and protein complex, are categorized into 2 major types: BOX C/D and BOX H/ACA [20]. Additionally, some RNPs exhibit cell specificity [21]. RNPs are involved in the modulation of DNA repair, telomeres, transcription processes, and RNA splicing [16]. Abnormal RNP expression or function usually results in disease occurrence, including carcinogenesis [22]. RNP expression also affects cancer cell proliferation and metastasis [23]. In terms of treatment, RNPs influence the antitumor efficacy of current therapies [24]. Additionally, RNPs are natural drug targets because of their RNA–RNA, RNA–protein, and protein–protein interactions and structural features. Consequently, novel RNP-based therapies are developing and assessed to treat cancer, paving the way for the clinical application of these drugs [25]. In the process, protein modification importantly mediates the interactions between cancer and RNPs, modulating cancer progression and treatment. Protein modification affects cancer progression through the regulation of protein stability, activation, and function [26,27]. For instance, in cancer cells, transforming growth factor-β (TGF-β) promoted hnRNP E1 SER43 phosphorylation to enhance the TGF-β-mediated epithelial-to-mesenchymal transition, leading to cancer metastasis [28]. Furthermore, combining knockdown of hnRNP L and oxaliplatin enhanced the phosphorylation levels of ATM and decreased the phosphorylation levels of 53BP1 and BRCA1, overcoming colorectal cancer oxaliplatin resistance [29]. Part of cancer-associated modifications targets granule-resident proteins, though their occurrence and function are not necessarily dependent on fully assembled granule structures. Protein modifications can occur within canonical membrane-less RNPs (such as stress granules, P-bodies, paraspeckles, and Cajal bodies), RNP complexes, or individual RNPs. This review will primarily focus on protein modifications taking place in these RNPs. Therefore, we tried to summarize and discuss the complex interactions between RNPs and various protein modifications in cancer initiation, progression, and treatment, facilitating the clinical translation of RNP-based therapies.

## Protein Modifications in RNPs

Protein modification is a crucial post-translational modification that regulates protein stability and function. Types of protein modification have been identified in RNPs, such as ubiquitination, phosphorylation, and SUMOylation, among others [30]. RNP granule modifications modulate RNP assembly by altering critical protein–protein and protein–RNA interaction affinities, changing the stability and function of RNPs [16]. The modified RNPs can mediate cancer progression. For example, in prostate cancer, the Cullin 3/SPOP E3 ligase complex induced hnRNP K ubiquitination and thus facilitated its degradation, attenuating cancer cell proliferation [31]. Furthermore, multiple protein modifications can occur within a single RNP granule. These modifications may compete within the same granule, where one modification can suppress another. These competitive protein modifications in RNPs differentially regulate cancer cells, influencing tumorigenesis and cancer progression. For example, in breast cancer, PCAT6, a long noncoding RNA (lncRNA), promoted hnRNPA2B1 ISGylation to prevent ubiquitination-mediated proteasomal degradation by binding to ISG15 [32]. Then, hnRNPA2B1 enhanced m6A-tagged mRNAs nuclear export via the ALYREF/NXF1 complex, leading to breast cancer stem cells’ stemness and doxorubicin resistance. In addition to direct modifications within RNPs, RNPs also regulate downstream protein modifications that influence cancer biology. For instance, RRP9, a specific component of the U3 snoRNP, inhibited the ubiquitination of JUN and thus stabilized it by down-regulating MDM2 [33]. Then, stabilized JUN stimulated the Ak strain transforming (AKT) pathway by activating AKT phosphorylation, promoting breast cancer progression. Exploring, discussing, and identifying these complex interactions within cancer and various protein modifications may be a promising approach to control cancer.

### Phosphorylation

Protein phosphorylation occurs through kinase-mediated covalent attachment of phosphoryl groups to specific amino acid residues (serine, threonine, or tyrosine), thereby modulating protein activity, localization, and interaction networks by altering structural conformations. Thus, phosphorylated protein can affect disease occurrence and progression, including cancer [34,35]. Phosphorylated RNPs also modulate tumorigenesis. For instance, HULC, an lncRNA, promoted YB1 (a major component of RNPs) phosphorylation, facilitating hepatocarcinogenesis [22]. Furthermore, phosphorylated RNPs are importantly involved in the regulation of cancer progression. For example, in prostate cancer, CSNK1D stabilized hnRNP A2B1 by up-regulating its phosphorylation levels to promote miR-25-3p/miR-93-5p maturation in an N^6^-methyladenosine (m^6^A)-dependent manner [36]. Subsequently, miR-25-3p depressed the FOXO pathway by targeting FOXO3. Furthermore, miR-93-5p stimulated the TGF-β pathway through the inhibition of BAMBI. Finally, these regulations facilitated prostate cancer cell proliferation and migration. Sui et al. [37,38] found that a DNA-dependent protein kinase catalytic subunit facilitated hnRNP A1 phosphorylation to promote RPA-to-POT1 switch during the G2 and M phases, facilitating cancer cell proliferation. Combined inhibition of ATR and PP2A would result in a catastrophic mitosis and synthetic lethality of tumor cells. In hepatocellular cancer, CDK2 facilitated LARP1 (La ribonucleoprotein domain family member 1) phosphorylation, improving cancer cell proliferation, migration, and invasion [39]. In colorectal cancer, the phosphorylated hnRNP A0 facilitated mitosis by activating RAS-associated RAB3GAP1-ZWINT1 cascade, leading to cancer cell proliferation [40]. In pancreatic ductal adenocarcinoma, AKT1/2-induced hnRNP L phosphorylation could interact with DDX17 to generate an alternative splicing complex that facilitated Caspase 9b and mH2A1.2 formation, leading to cancer proliferation and metastasis [41]. Up-regulation of tRF-21 expression depressed AKT1/2-induced hnRNP L phosphorylation, reversing cancer progression. In another pancreatic cancer research, Fyn enhanced the phosphorylation levels of hnRNP E1 by activating PAK1, facilitating cancer metastasis [42]. These results indicate that phosphorylated hnRNPs promote cancer cell proliferation, invasion, and metastasis. Blocking hnRNP phosphorylation inhibits hnRNP-induced cancer progression. It notices that targeting the phosphorylation of hnRNPs may be a promising way to manage cancer cells.

**Fig. 1.**
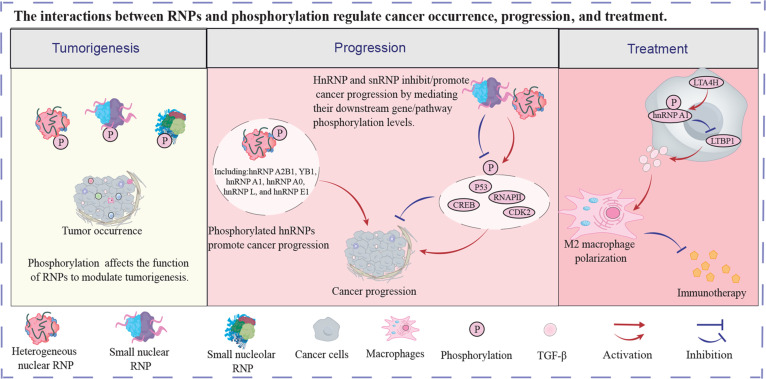
The interactions between RNPs and phosphorylation regulate cancer occurrence, progression, and treatment. Different RNPs exhibited anti-/protumor function. RNP, ribonucleoprotein.

Furthermore, RNPs can modulate cancer progression by affecting the phosphorylation levels of downstream genes. For example, SF3B1, a U2 snRNP factor, interacted with phosphorylated RNAPII to increase cancer proliferation, while impairing the interaction by the CDK12/13 inhibitor THZ531 would lead to cancer inhibition [43]. In lung cancer, knockdown of hnRNP A2/B1 induced CDK2 and P53 phosphorylation, cyclin E degradation, and P21 and P27 expression, but depressed ERK1/2 phosphorylation [44]. Consequently, knockdown of hnRNP A2/B1 resulted in cell cycle arrest, blocking cancer cell proliferation. In another lung study, hnRNP A1 enhanced CCND1 expression by promoting VRK1-mediated phosphorylation of CREB, increasing cancer cell proliferation [45]. The above results indicate that hnRNPs and snRNPs can also facilitate cancer proliferation and metastasis by elevating the phosphorylation levels of relative proteins. Additionally, hnRNP expression and phosphorylation was also involved in the regulation of the immune TME, which importantly affects treatment efficacy [46]. In hepatocellular carcinoma, LTA4H improved hnRNP A1 phosphorylation, impairing LTBP1 expression [47]. Knockout of LTA4H in cancer cells enhanced TGF-β generation and secretion by recovering LTBP1 expression, leading to M2 macrophage polarization. The M2 macrophage accumulation resulted in immune evasion and immunotherapy resistance. These studies demonstrate that reducing phosphorylation levels of RNPs and related proteins substantially suppresses cancer progression, even without directly targeting these molecules. Thus, it is important to explore and identify the interactions between phosphorylation and RNPs. Figure 1 shows the regulatory network of the above results.

### Ubiquitination

Ubiquitination is a post-translational modification mediated by E1, E2, and E3 ligases, which covalently attaches ubiquitin to lysine residues on target proteins, forming polyubiquitin chains to regulate protein degradation, DNA repair, signal transduction, and cellular homeostasis [48,49]. Ubiquitination affects cancer progression by mediating protein stability and function, including RNPs. For example, DRAIC, a circRNA, prevented hnRNP A2B1 from FBXO11-mediated ubiquitination and proteasome-dependent degradation, destabilizing m6A-modified IGF1R and depressing clear cell renal carcinoma progression [50]. In colorectal cancer, CRNDE protected hnRNP A2B1 from TRIM21-mediated K63 ubiquitination-dependent degradation, enhancing KRAS expression [51]. Then, overexpression of KRAS activated the mitogen-activated protein kinase (MAPK) pathway to promote cancer progression. Similarly, Linc01232 protected hnRNP A2B1 from ubiquitination and ubiquitination-induced degradation [52]. The stabilized hnRNP A2B1 facilitated pancreatic cancer metastasis via the A-Raf-induced MAPK/ERK pathway. The above results identify that up-regulation of hnRNP A2B1 stability results in cancer cell growth and metastasis by preventing ubiquitination-induced protein degradation. Subsequent studies found that hnRNP A2B1 degradation induced by ubiquitination significantly promoted cancer progression. In hepatocellular carcinoma, miR503 promoted ubiquitination-induced hnRNP A2B1 degradation to activate the NF-κB pathway, suppressing cancer progression [53]. Another hepatocellular carcinoma study indicated that FBXO11 bound to hnRNP A2B1 and induced its ubiquitination-mediated degradation, blocking cancer cell progression [54]. CAND1 suppressed the SCF/FBXO11 complex to improve lipid generation and accumulation, facilitating cancer cell proliferation and metastasis. Both gain- and loss-of-ubiquitination studies demonstrate that stable expression of hnRNP A2B1 promotes cancer progression, while ubiquitination-mediated degradation of hnRNP A2B1 effectively suppresses tumor growth and metastasis. This suggests that targeting hnRNP A2B1 or enhancing its ubiquitination may represent a promising therapeutic strategy against cancer.

Furthermore, many other RNPs also interact with ubiquitination to affect cancer cell biology. R-loops are substantially involved in cancer progression and treatments [55]. In ovarian cancer, PLADE, an lncRNA, promotes hnRNP D degradation through von Hippel-Lindau tumor suppressor protein-mediated ubiquitination, improving R-loop generation and accumulation and consequently improving cisplatin sensitivity in cancer cells [56]. In another ovarian cancer study, FBXO16 significantly enhanced hnRNP L ubiquitination and ubiquitination-induced degradation by interacting with the RRM3, depressing cancer cell proliferation [57]. In lung cancer, circZFR, a circRNA, depressed hnRNP LL ubiquitination to prevent its degradation, enhancing oxidative phosphorylation in cancer cells [58]. Subsequently, hnRNP LL up-regulated the AKT/mTOR pathway by elevating MYO1B splicing, leading to lung cancer progression. In pancreatic ductal adenocarcinoma, cNEK6, a circRNA, blocked BTRC-induced K48 ubiquitination in small RNP peptide A, preventing its degradation [59]. Subsequently, small RNP peptide A bound to the G-quadruplex structures in PP2Ac, activating the mTROC1 pathway and thus resulting in glycolysis-induced gemcitabine resistance. SHP2D^61Y^ and SHP2^E76K^ mutations in colorectal cells enhanced glycolysis and lactate production, inducing cisplatin resistance and cancer metastasis [60]. Mechanically, SHP2^D61Y^ and SHP2^E76K^ mutations evoked PKM2 expression, preventing hnRNP K ubiquitination and consequently leading to cancer progression. These results demonstrate that ubiquitination is involved in cancer progression and treatments by modulating many RNPs. Moreover, RNPs also mediated the ubiquitination levels of its relative proteins to affect cancer growth. For instance, LINC00662 stabilized by hnRNP H1 could protect GRP78 from ubiquitination and ubiquitination-induced degradation, activating the P38/MAPK pathway and leading to ovarian cancer cell progression [61]. Additionally, ubiquitination also mediates SNRNP stability. For instance, tiRNA-MET, a tRNA-derived fragment, could combine with the RRM2 domain of snRNP A, leading to snRNP A ubiquitination and ubiquitination-induced degradation [62]. The down-regulation of snRNP A resulted in breast cancer inhibition via the mTORC1 pathway. Collectively, cancer cells may be effectively depressed by sustaining a proper ubiquitination level in the regulatory network of RNPs.

Additionally, the interactions between ubiquitination and RNPs are involved in the TME modulation. In regulatory T cells, hnRNP A1 protected FOXP3 from STUB1-mediated ubiquitination, sustaining the immune modulative function of regulatory T cells [63]. In cancer-associated fibroblasts, USP7 could attenuate ubiquitination-induced hnRNP A1 degradation [64]. Subsequently, hnRNP A1 promoted miR-522 packing into exosomes, which depressed ALOX15 expression in gastric cancer cells, leading to chemotherapy resistance by attenuating lipid-reactive oxygen species accumulation. Exosomes containing miR-3153 from lung cancer cells could prevent MINK1 ubiquitination by targeting ZFP91 and thus induced the JNK pathway activation in macrophages, promoting macrophage M2 polarization [65]. During this process, hnRNP A2B1 enhanced miR-3153 generation and secretion in cancer cells, leading to lung cancer progression.

**Fig. 2.**
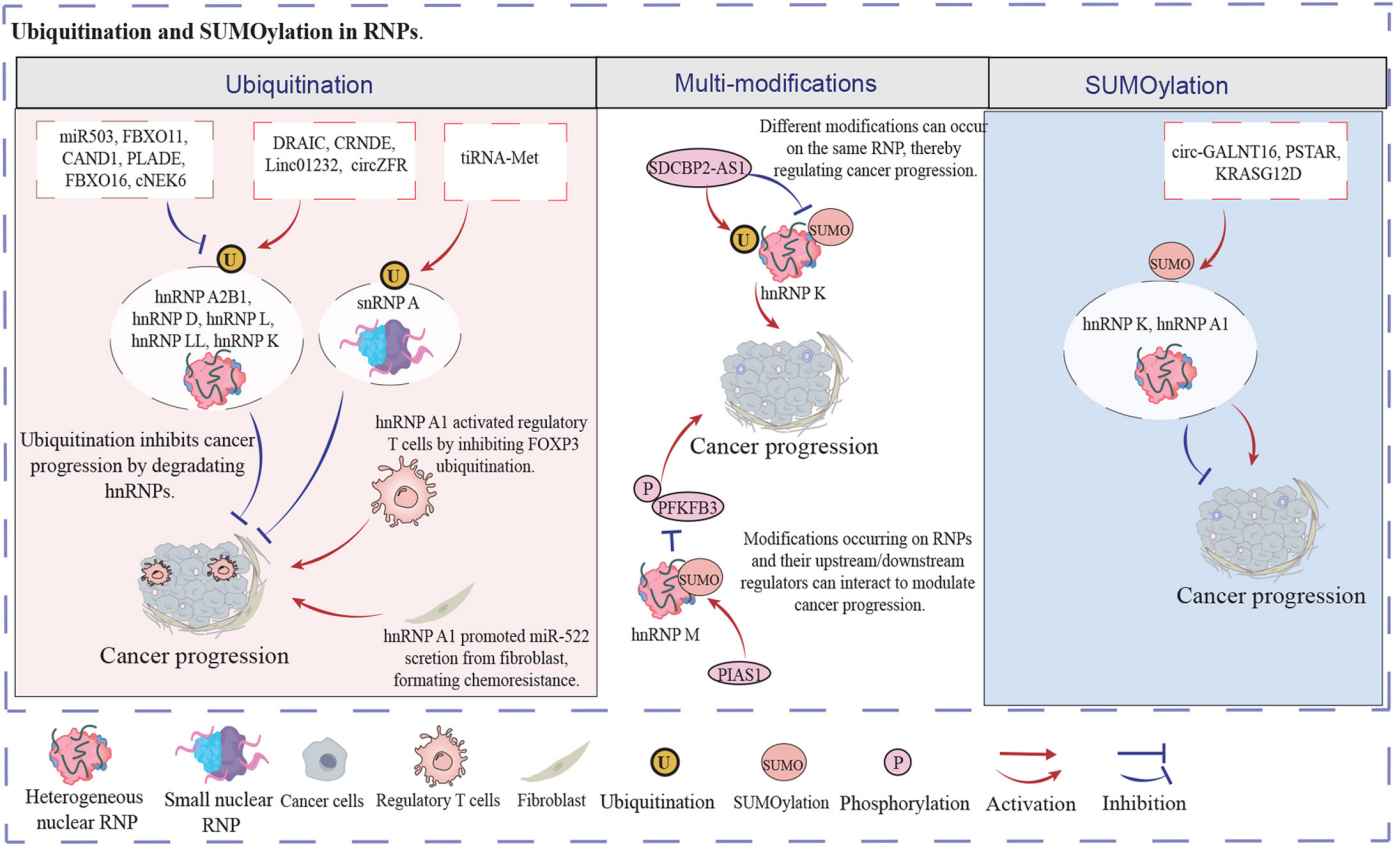
Ubiquitination and SUMOylation in RNPs. Ubiquitinated RNPs can usually be degraded, diminishing cancer progression. Protein modifications can competitively occur on the same RNP granule, affecting the RNP granule expression and function. SUMOylated RNPs can positively or negatively cancer progression according to the down-stream pathway of the RNP.

These results demonstrate that RNP granule-regulated ubiquitination levels modulate immune cell function. In cancer and stromal cells, RNP granule-mediated ubiquitination influences intercellular crosstalk by regulating exosome secretion, suggesting its potential role in remodeling the TME and providing a rationale for immunotherapeutic targeting. Figure 2 exhibits above regulatory mechanism.

### SUMOylation

SUMOylation is a post-translational modification process mediated by a cascade of E1, E2, and E3 ligases, which conjugates SUMO proteins to target substrates, regulating subcellular localization, protein stability, interaction networks, and critical cellular processes such as transcriptional control, genome integrity, and stress responses [66,67]. The interactions between hnRNPs and SUMOylation importantly influence cancer proliferation and metastasis. For instance, a colorectal cancer study found that circ-GALNT16 bound to the KH3 domain of hnRNP K, enhancing its SUMOylation by blocking SENP2-mediated hnRNP K deSUMOylation and promoting hnRNP K/P53 complex formation [68]. Elevation of the hnRNP K/P53 complex impaired cancer cell progression. Similarly, PSTAR, an lncRNA, could enhance hnRNP K SUMOylation, improving the interaction between P53 and hnRNP K and consequently impairing hepatocellular cancer progression [69]. P53 is a famous antitumor gene. These 2 researches proved that SUMOylation facilitated the hnRNP K/P53 complex formation to depress cancer cell progression. Furthermore, in bladder cancer, SUMOylated DDX39B enhanced circNCOR1 nuclear exportation, depressing lymphangiogenesis and lymph node metastasis [70]. Mechanically, circNCOR1 in the cancer cell nuclear interacting with hnRNP L improved H3K9 acetylation in the promoter of SMAD7 to down-regulate the TGFβ/SMAD pathway, impairing bladder cancer lymph node metastasis. Consequently, down-regulation of circNCOR1 in the cancer cell nuclear would reverse the TGFβ/SMAD pathway inhibition, promoting cancer cell lymph node metastasis. However, in pancreatic cancer, KRASG12D promoted hnRNP A1 SUMOylation in extracellular vesicle to stabilize PROX1 mRNA in lymphatic endothelial cells, resulting in lymphangiogenesis and cancer cell lymph node metastasis in vitro and in vivo [71]. These conflicting findings indicated that SUMOylation of either hnRNPs or hnRNP-regulated proteins can modulate tumor lymph node metastasis. These results suggest that SUMOylated RNPs exhibit antitumor and protumor function. Consequently, we propose that RNP granule SUMOylation may represent a potential mechanism for inhibiting tumor progression and metastasis through protein modification.

Moreover, there are different protein modifications in RNPs [72]. For instance, in gastric cancer, SDCBP2-AS1 expression promoted the ubiquitination of and decreased SUMOylation of hnRNP K, leading to β-catenin degradation and thus suppressing cancer cell metastasis [73]. This research identified that the balance of ubiquitination and SUMOylation levels in RNPs significantly affect cancer cell progression. Beyond competitive interactions, protein modifications also engage in hierarchical cascades in RNPs. For example, PIAS1-induced SUMOylated hnRNP M could diminish cancer cell glycolysis by inhibiting PFKFB3 phosphorylation, depressing colorectal cancer occurrence and progression [74]. Glycolysis in cancer cells would reverse when hnRNP M was de-SUMOylated by SENP1, recovering the progression of cancer cells. In hepatocellular cancer, high H3/H4 histone acetylation levels were observed in the promoter of ANCR (an lncRNA) [75]. Subsequently, ANCR blocked the degradation of hnRP A1 by inhibiting the ubiquitination levels of hnRNP A1 in vitro and in vivo, leading to cancer proliferation and metastasis. The protein modifications of RNPs are highly complex, involving not only competing modifications within individual granules but also regulatory effects on downstream or associated proteins. Thus, deciphering RNP granule modification patterns and their functional consequences is critical for developing modification-targeted cancer therapies. Figure 2 shows the regulatory network of the above results.

### Acetylation

Acetylation is a post-translational modification mediated by acetyltransferases and reversed by deacetylases, which covalently attaches acetyl groups to lysine residues, modulating chromatin structure, transcriptional activity, protein interactions, and metabolic regulation [76,77]. Acetylation importantly influences cancer occurrence, progression, and treatments [78]. For example, KHSRP, the hnRNP K-homology splicing regulatory protein, could be acetylated by androgen, impairing DNA damage response and leading to prostate tumorigenesis [79]. In terms of cancer progression, ESCO2 acetylated hnRNP A1 at K277 and thus inhibited hnRNP A1 nuclear export in lung cancer [80]. Subsequently, the acetylated hnRNP A1 could inhibit PKM1 isoform formation and promote PKM2 isoform formation by binding to the EI9 of PKM mRNA, improving aerobic glycolysis in cancer cells and consequently resulting in cancer cell proliferation. In lung cancer, hnRNPA2/B1 acetylated by P300 effectively promoted COX2 expression, leading to cancer cell proliferation [81]. These studies showed us that hnRNPs could be directly acetylated and thus affected cancer cell proliferation. Furthermore, acetylation usually occurs in histone proteins [77]. RNPs also regulate cancer progression by interacting with acetylated histone proteins. For instance, in colorectal cancer, c-MYC increased the histone acetylation levels in the promoter of CASC11 (an lncRNA) [82]. Subsequently, overexpression of CASC11 activated the WNT/β-catenin pathway by targeting hnRNP K, resulting in cancer cell proliferation and metastasis in vitro and in vivo. In cancer cells, hnRNP A2 activated by mitochondrial stress could elevate the acetylation levels of histone H4 at K8 on target gene, resulting in cancer progression [83]. RNPs themselves undergo acetylation and can modulate the acetylation of associated proteins. Current studies indicate that acetylated RNPs promote tumorigenesis and progression in a context- and cell-type-dependent manner, suggesting that therapeutic targeting of RNP granule acetylation may represent a promising strategy for cancer intervention.

### Methylation

Methylation usually adds methyl groups to lysine/arginine residues (proteins) or cytosine (DNA), regulating chromatin remodeling, transcriptional silencing, protein stability, and cellular signaling pathways [84,85]. RNPs facilitate cancer cell growth by inducing methylation. For instance, PRMT5 enhanced snRNP D3 protein methylation via its cTD4 domain, facilitating snRNP D3–TDRD1 interactions in the cytoplasm and consequently promoting prostate cancer proliferation [86]. In another cancer study, arginine methylation in hnRNP K would disrupt the hnRNP K–DDX3 interaction, impairing DDX3-induced cancer cell apoptosis [87]. Moreover, RNPs are also involved in cancer growth by affecting downstream protein methylation. In bladder cancer stem cells, lncLBCS facilitated the hnRNP K–EZH2 complex formation by serving as a scaffold [88]. Subsequently, the hnRNP K–EZH2 complex promoted H3K27 tri-methylation to depress SOX2 expression, depressing cancer cell self-renewal and chemoresistance. In hepatocellular cancer, hnRNP U depressed H3K27 tri-methylation and enhanced H3K27 and H3K9 acetylation to activate CDK2 expression, leading to cancer cell proliferation [89]. RNPs can undergo methylation and regulate methylation of associated proteins, but their context-dependent roles in tumor progression require further experimental validation.

### Crotonylation

**Fig. 3.**
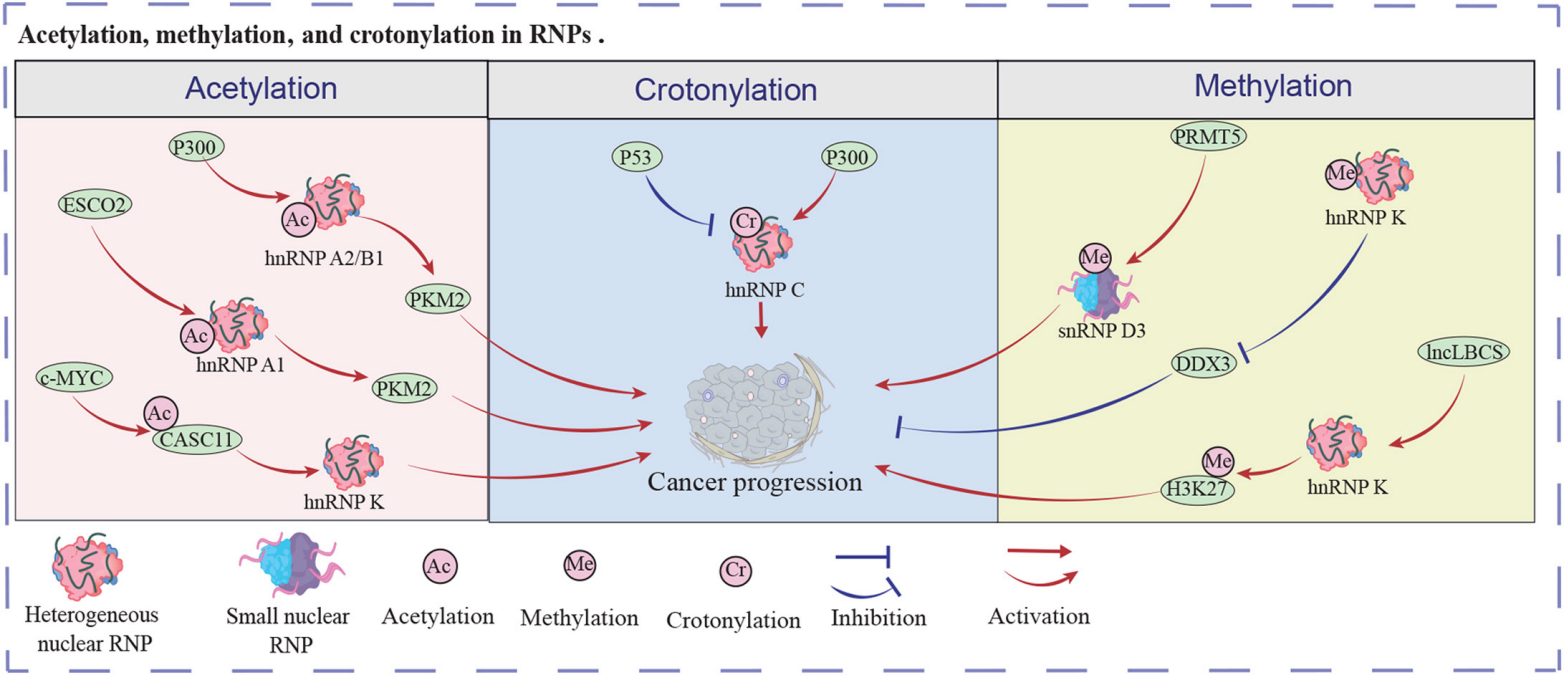
Acetylation, methylation, and crotonylation in RNPs. These modifications could directly occur on RNPs to modulate cancer progression.

Crotonylation is a post-translational modification mediated by crotonyltransferases and decrotonylases, which attaches crotonyl groups to lysine residues, regulating chromatin dynamics, transcriptional activation, and metabolic reprogramming, particularly in spermatogenesis and cancer progression [90,91]. For example, p53 deficiency or mutation in cancer cells improved CCND1 and MCM3 mRNA stability via the MDM2/HDAC3 axis by increasing the expression and crotonylation level of hnRNP C, promoting colorectal cancer progression [92]. In another colorectal cancer research, the PTBP1 crotonylation level significantly enhanced the PKM2/PKM1 ratio by interacting with hnRNP A1/2 [93]. Subsequently, the PKM alternative splicing increased glycolysis and lactic acid production in cancer cells, promoting colorectal cancer cell progression. Furthermore, knockdown p300-diminished the lysine crotonylation levels of hnRNP A1 expression in HeLa cells [94]. The decreased hnRNP A1 crotonylation level resulted in cervical cancer cell inhibition. Current findings demonstrate that crotonylation regulates tumor growth and metastasis by modulating RNPs or its up- or downstream proteins. Figure 3 exhibits above regulatory mechanism.

## Other Protein Modifications

Glycosylation is an enzymatic process that covalently attaches glycans to proteins or lipids, regulating their structure, stability, trafficking, and molecular interactions critical to cellular function [95,96]. Glycosylation levels substantially influence cancer progression and treatments [97]. Currently, studies reported that RNPs could affect the glycosylation levels of relative proteins, mediating cancer progression. For example, human antigen R (HuR), an RNP granule, induced immune evasion in the breast TME by binding and stabilizing PD-L1 mRNA [98]. Niclosamide, a HuR translocation inhibitor, could reverse the antitumor function of T cells by abolishing the PD-L1 glycosylation level, improving immunotherapy efficacy. In another study, METTL3 and hnRNP U promoted the generation and maturation of miR-151-5p through the up-regulation of m^6^A modification, depressing LYPD3 expression and the progression of head and neck squamous cell carcinoma [99]. Down-regulation of miR-151-5p or elevation of the glycosylation levels of LYPD3 significantly diminished cancer cell proliferation and invasion. These 2 studies illustrated that RNPs modulated cancer progression and the immune TME by affecting its relative protein glycosylation levels. However, a few cancer studies report that glycosylation occurs on RNPs. O-GlcNAcylation, one type of glycosylation, that occurred on hnRNP K could promote cholangiocarcinoma progression [100]. Consequently, the direct interactions between glycosylation and RNPs remain unclear and need to be explored in future work.

Some modifications are also involved in the mediation of RNPs. For instance, fucosylation can influence disease occurrence and progression, including cancer [101,102]. In melanoma, fucosylated RPS3 exhibited enhanced binding affinity for hnRNP U than un-fucosylated RPS3, promoting tumor progression [103]. HIFAL, a HIF-1α antisense lncRNA, could recruit PHD3 to PKM2, enhancing PKM2 hydroxylation levels [104]. Subsequently, the PKM2/PHD3 complex elevated HIF-1α transactivation by binding to hnRNP F, leading to breast cancer progression in vivo. SNORA73 depressed PARP1 auto-PARylation by formatting a noncanonical snoRNP, impairing the occurrence of acute myeloid leukemia [105]. Recent studies reveal that multiple protein modifications regulate tumor growth and metastasis by targeting RNP granule-associated proteins, yet whether these modifications occur directly on RNPs themselves remains unexplored and requires systematic investigation. Figure 3 shows the regulatory network of the above results. Table S1 shows the key reference of this work.

## Discussion and Perspectives

Protein modifications substantially influence cancer occurrence, progression, and treatments by modulating RNPs or its relative proteins. Studies have shown that RNPs can directly undergo phosphorylation, ubiquitination, SUMOylation, methylation, crotonylation, and acetylation, but no studies have reported glycosylation, fucosylation, or PARylation on RNPs. These modifications can competitively occur on RNPs. Cancer cells, immune cells, and stromal cells can undergo RNP granule modifications. These results provide the basis of RNP granule-based antitumor therapy. However, we have to note that further steps are required before clinical application.

Firstly, studies should identify specific modification sites on RNPs. Some researches report the specific modification sites of RNPs [80,81]. It gives researchers specific information to explore and discuss the interactions among various modifications. The specific modification sites also allow researchers to design novel drugs. However, many current studies do not explore or report the specific modification sites of RNP granule modifications. This may lead to difficulties in understanding RNP granule modifications and experimental design challenges, creating knowledge gaps in modification interactions that hinder drug development. Furthermore, multiple protein modifications can competitively occur on a single RNP [73,74]. The dynamic balance formed by this competition critically influences tumor initiation and progression. Therefore, modulating this competitive equilibrium may represent a key therapeutic strategy for targeting tumors through RNP regulatory mechanisms. Recently, proteolysis-targeting chimera (PROTAC) appears to be a promising approach to manage cancer cells by targeting protein degradation via the endogenous ubiquitin proteasome system [106]. Therefore, identifying the precise binding site provides a crucial foundation for developing PROTAC-based therapeutics. We strongly recommend that future studies report specific modification sites to facilitate further research and therapeutic design. Furthermore, current research on protein modifications predominantly focuses on the “writing” phase, with limited exploration of the “erasing” and “recognition” processes within RNP and tumors, except for phosphorylation, which has been relatively well-studied [107]. Thus, the next work needs to explore these processes in the protein modification of RNPs within cancers. Moreover, as previously mentioned, RNP granules are RNP particles assembled through RNA–RNA, protein–protein, and protein–RNA interactions [16]. Therefore, in cancer cells, whether protein modifications affect the composition of specific RNPs, and whether they alter the intermolecular interactions within these granules should be determined. Understanding these dynamic changes will provide a solid foundation for RNP granule-based drug development.

Furthermore, the TME includes cancer cells, immune cells, and stromal cells [108,109]. The interactions between cancer cells and other cells substantially influence cancer progression [110]. RNP granule modifications in cancer cells can also affect immune function, regulating the immune TME. In hepatocellular carcinoma, LTA4H improved hnRNP A1 phosphorylation, impairing LTBP1 expression [47]. Knockout of LTA4H in cancer cells enhanced TGF-β generation and secretion by recovering LTBP1 expression, leading to M2 macrophage polarization, resulting in immune evasion and immunotherapy resistance. Various cells in the TME may generate different reactions under the same stimulation [111,112]. As mentioned above, RNPs exhibit cell-type specificity, raising the question of whether identical modifications in different cell types lead to distinct functional alterations [21]. In cancer cells, SUMOylated hnRNPs significantly depressed cancer proliferation and metastasis [68,69]. However, in lymphatic endothelial cells, SUMOylated hnRNP A1 could stabilize PROX1 mRNA, resulting in lymphangiogenesis and cancer cell lymph node metastasis in vitro and in vivo [71]. Therefore, investigating protein modifications in RNPs within individual cells can further evaluate the impact of RNP granule-based drugs on the TME, providing both theoretical and experimental foundations for clinical applications.

Recently, the U.S. Food and Drug Administration suggests that animal testing should be replaced by other models to assess drug efficacy and toxicity [113]. Human organoids and organ-on-a-chip systems are deemed as alternative approaches to promote drug development [114,115]. Artificial intelligence (AI)-based computational models are tools to assess the toxicity of drugs [116,117]. These tools can also be used to explore the protein modifications of RNPs. Other experiments, such as “granule rescue” experiment [118] and granule imaging [119], can also promote RNP research progression. Many kinds of mass spectrometry can also be used to explore RNPs [120]. Furthermore, the newly established single-cell proteomics approach enables the investigation of RNP protein modifications across individual cells, thereby addressing limitations in studying RNP cell specificity [121].

## Ethical Approval

The authors are accountable for all aspects of the work in ensuring that questions related to the accuracy or integrity of any part of the work are appropriately investigated and resolved.
