# Supplementary material for: Harnessing Ribonucleoprotein Granule Biology for Cancer Therapy: The Central Role of Protein Modifications
Source: Research (Wash D C). 2026 Feb 18;9:1133. doi: 10.34133/research.1133 (PMC12914060; doi:10.34133/research.1133)
Supplement: Supplementary 1 — Table S1 [file research.1133.f1.docx]

**Supplementary Table 1.** The key references.

| **Supplementary Table 1 \|** The key references. | | | | | | | |
| --- | --- | --- | --- | --- | --- | --- | --- |
| **PM types** | **Cancer** | **Ribonucleoprotein** | | **Cell line** | **Animal** | **Pathway/gene** | **PMID** |
| Glycosylation | Breast | | HuR | MDA-MB-231, SUM159, EMT6, MIA PaCa-2, H460, LL/2, A549, 293-FT and HeLa. | Female BALB/c mice and C57BL/6 mice. | PD-L1. | 37848943[98] |
| Glycosylation | Head and neck | | hnRNP U | NOK, HOK, HEK293T, HN4, HN12, HN30, HN31, 1386Tu, 1386Ln, SCC25 and Cal27. | Female BALB/c nude mice. | The METTL3/miR-151-5p/LYPD3 axis. | 39009906[99] |
| Fucosylation | Melanoma | | hnRNP U | 293T, and NALM6. | Mice. | RPS3. | 34070332[103] |
| SUMOylation | Colorectal | | hnRNP M | 293T, HCT116, MC38, SW620 and HT29. | BALB/c-nude mice. | PFKFB3, SENP1 and PIAS1. | 39983892[74] |
| SUMOylation | Gastric | | hnRNP K | HEK293T, AGS, GES-1, BGC823, SGC7901, MGC803, HGC27, and N87. | Female BALB/c nude mice. | SDCBP2-AS1. | 36209503[73] |
| SUMOylation | Pancreatic | | hnRNP A1 | PANC-1, AsPC-1, Capan-2, Mia-PaCa2 and BxPC-3. | Four-week-old nude mice. | PROX1 and KRASG12D. | 35579947[71] |
| SUMOylation | Bladder | | hnRNP L | SV-HUC-1, MB49, T24, UM-UC-3, RT112 and UM-UC-1. | BALB/c nude mice and C57BL/6 mice. | DDX39B/circNCOR1/SMAD7 axis. | 35395674[70] |
| SUMOylation | Colorectal | | hnRNP K | LoVo, DLD-1, SW-480, Caco-2, RKO, HCT 116, HT-29, and NCM460. | Male BALB/c nude mice. | PTBP1. | 39497094[93] |
| Crotonylation | Colorectal | | hnRNP A1/2 | HEK293T, HCT116, SW620, and SW480. | Male BALB/c nude mice. | P53. | 31148184[69] |
| Crotonylation | Cervical | | hnRNP A1 | HCerEPiC, HeLa, Caski and SiHa. | / | p300. | 33457194[94] |
| Crotonylation | Colorectal | | hnRNP C | HCT116 and WSU-HN6. | BALB/c nude mice. | The p53/MDM2/HDAC3/hnRNP C axis. | 40482911[92] |
| Acetylation | Prostate | | hnRNP K | 293T, LNCaP, 22RV1 and DU145. | Male NOD-SCID mice. | KHSRP. | 38501452[79] |
| Acetylation | Lung | | hnRNP A1 | A549 and NCI-H1975. | NOD-SCID mice. | ESCO2. | 33573689[80] |
| Acetylation | Hepatocellular | | hnRNP A1 | LO2, Hep3B, HepG2 and Huh7. | Female nude mice. | ANCR. | 31868085[75] |
| Acetylation | Lung | | hnRNPA2/B1 | H1299, H322, HLF and HB. | Female nude mice. | COX2. | 26774881[81] |
| Acetylation | Colorectal | | hnRNP K | FHC, LOVO, M5, HCT116, HT29, RKO, LS174T, SW480, SW620, and HEK293. | Male athymic BALB/c nude mice. | CASC11. | 27012187[82] |
| Ubiquitination | Breast | | RRP9 | BT549, SK-BR-3, MCF-10 A, BT-474, MDA-MB-231 and MCF-7. | BALB/c nude mice. | JUN. | 39702367[33] |
| Ubiquitination | Pancreatic | | snoRNP A. | PANC-1 and PaTu8988t. | Male athymic BALB/c nude mice. | cNEK6. | 39394197[59] |
| Ubiquitination | Colorectal | | hnRNP K | HT-29, SW480, VACO432 and HCT116. | Female BALB/c nude mice. | PKM2 and SHP2. | 39104405[60] |
| Ubiquitination | Renal | | hnRNP A2B1 | 293 T, HK2, Caki-1, 769-P, 786-O and ACHN. | BALB/c Nude mice. | DRAIC/hnRNPA2B1/FBXO11/IGF1R axis. | 38811846[50] |
| Ubiquitination | Breast | | HnRNP A2B1 | BT‐549, Hs578T, MDA‐MB‐231 and MCF‐7. | Female nude mice. | ALYREF/NXF1 Complex. | 38626369[32] |
| Ubiquitination | Ovarian | | hnRNP D | SKOV3 and COV504. | Mice. | PLADE. | 38225339[56] |
| Ubiquitination | Hepatocellular | | hnRNP A2B1 | HEK293T, Hep3B, Huh7, HCCLM3, HepG2, Hep 1−6 and PLC5. | Male BALB/c‐nu mice. | the CAND1/SCFFBXO11 /hnRNPA2B1 axis. | 37837399[54] |
| Ubiquitination | Colorectal | | hnRNP A2B1 | DLD1, SW480, SW620, HCT116, HT29 and RKO. | BALB/c nude mice. | CRNDE. | 37716979[51] |
| Ubiquitination | Lung | | hnRNP LL | A549, HCC827, H1975 and PC9. | Female BALB/c nude mice. | circZFR. | 37461053[58] |
| Ubiquitination | Lung | | hnRNP A2B1 | BEAS-2B, THP-1, HEK-293T, A549 and Calu3. | BALB/c nude mice. | miR-3153. | 37010098[65] |
| Ubiquitination | Prostate | | hnRNP K | HEK293, HEK293T, MEFs PC-3, DU145, 22Rv1, C42, VCaP and LNCaP. | BALB/c-nu/nu mice. | SPOP. | 34857003[31] |
| Ubiquitination | Ovarian | | hnRNP L | HEK293T, A2780, OVCAR8 and SKOV3. | Male BALB/cA nude mice. | FBXO16. | 34333526[57] |
| Ubiquitination | Ovarian | | hnRNP H1 | TOV21G, OVCAR-3, CAOV-3, SK-OV-3 A2780, OVCA429, OVCAR433, OVCAR-4 and ES-2. | Female athymic BALB/c nude mice. | LINC00662. | 34148056[61] |
| Ubiquitination | Pancreatic | | hnRNP A2B1 | HPNE, BXPC-3, COLO-357, CFPAC-1, MIAPACA-2 and PANC-1. | Male BALB/cA-nu mice. | The A-Raf-induced MAPK/ERK pathway. | 32814086[52] |
| Ubiquitination | Gastric | | hnRNP A1 | SGC7901, MGC803 and MKN45. | Male nude mice. | The USP7/hnRNPA1 axis. | 32106859[64] |
| Ubiquitination | Hepatocellular | | hnRNP A2B1 | L02, 293T, SMMC-7721 and Huh7. | / | miR503. | 29774077[53] |
| Ubiquitination | Breast | | SNRNPA | MDA-MB-231 and BT-549. | Female BALB/c nude mice. | The mTORC1 pathway. | 40410734[62] |
| Methylation | Prostate | | snoRNP D3 | VCaP, HeLa, 293T, 22Rv1 and LNCaP. | NSG mice. | TDRD1. | 37041411[86] |
| Methylation | Bladder | | hnRNP K | HEK-293T, UM-UC-3, 5637, HT-1376, and J82. | Male BALB/c nude mice. | H3K27. | 30397178[88] |
| Methylation | Osteosarcoma | | hnRNP K | U2OS. | / | DDX3. | 34575922[87] |
| Methylation | Hepatocellular | | hnRNP U | MHCC97H, HEK293T, HepG2 and Huh7. | Female BALB/c nude mice. | CDK2. | 34737140[89] |
| Phosphorylation | Hepatocellular | | hnRNP A1 | Hepa1-6 and HepG2. | C57L/J mice. | The LTBP1/TGF-β axis. | 40056904[47] |
| Phosphorylation | Various | | SF3B1 | MDA-MB-231, OVCAR-3, MiaPaCa-2 and HEK293T. | / | RNAPII. | 37026485[43] |
| Phosphorylation | Prostate | | hnRNP A2B1 | RWPE-1, PC3 and DU145. | Male BALB/C nude mice. | CSNK1D. | 37208565[36] |
| Phosphorylation | Various | | hnRNP A1 | HCT116 and A549. | Athymic nude mice. | PP2A. | 34933911[38] |
| Phosphorylation | Hepatocellular | | LARP1 | Huh7, HepG2 and Hep3B. | C57BL/6 mice. | CDK2. | 34449924[39] |
| Phosphorylation | Pancreatic | | hnRNP L | Capan-2, SW1990 and 293T. | Female BALB/c nude mice and NOG mice. | tRF-21. | 34779408[41] |
| Phosphorylation | Lung | | hnRNP A2B1 | NCI-H358, A549, NCI-H1703 and NCI-H460. | / | The ERK/p53/HDM2 pathway. | 34109989[44] |
| Phosphorylation | Lung | | hnRNP A1 | A549, H1299 and HEK293A. | / | VRK1. | 34071140[45] |
| Phosphorylation | Colorectal | | hnRNP A0 | SUIT-2, HCT116, MKN45, PANC-1 and OE33. | Male BALB/c nude mice. | RAB3GAP1-ZWINT1. | 32303675[40] |
| Phosphorylation | Colorectal | | hnRNP L | SW480, SW620, HCT116, and HT29. | / | 53BP1 and BRCA1. | 31320608[29] |
| Phosphorylation | Pancreatic | | hnRNP E1 | AsPC1, BxPC3, CFPAC1, Panc1 and HEK293. | Male athymic nu/nu mice. | Fyn. | 28560430[42] |
| Phosphorylation | Various | | hnRNP E1 | MDA231, MDA435, MDA453, MDA468, SW480, SW620, HCT8, HCT116, HT29, CaCO_2,_ NMuMG, 67NR, 4TO7 and 4T1. | / | TGFβ. | 27067543[28] |
| Hydroxylation | Breast | | hnRNP F | MDA-MB-231, MCF-7, 76 N, 293 T, T47D, SK-BR3, BT549 and MDA-MB-468. | Balb/c nu/nu mouse. | HIF-1α and PKM2/PHD3 complex. | 33637716[104] |

OXPHOS: oxidative phosphorylation;

Kyn: Kynurenine;

ROS: reactive oxygen species;

NK: natural killer;
